# Genome-Wide Identification, Expression Profile and Evolution Analysis of Karyopherin β Gene Family in *Solanum tuberosum* Group Phureja DM1-3 Reveals Its Roles in Abiotic Stresses

**DOI:** 10.3390/ijms21030931

**Published:** 2020-01-31

**Authors:** Ya Xu, Lu Liu, Pan Zhao, Jing Tong, Naiqin Zhong, Hongji Zhang, Ning Liu

**Affiliations:** 1 College of Plant Protection, Yunnan Agricultural University, Kunming 650201, China; 2 State Key Laboratory of Plant Genomics, Institute of Microbiology, Chinese Academy of Sciences, Beijing 100101, China; 3 National Engineering Research Center for Vegetables, Beijing Vegetable Research Center, Beijing Academy of Agricultural and Forestry Sciences, Beijing 100097, China; 4 The Enterprise Key Laboratory of Advanced Technology for Potato Fertilizer and Pesticide, Inner Mongolia Autonomous Region, Hulunbuir 021000, China

**Keywords:** karyopherin, solanum tuberosum, abiotic stress, expression analysis

## Abstract

In eukaryotic cells, nucleocytoplasmic trafficking of macromolecules is largely mediated by Karyopherin β/Importin (KPNβ or Impβ) nuclear transport factors, and they import and export cargo proteins or RNAs via the nuclear pores across the nuclear envelope, consequently effecting the cellular signal cascades in response to pathogen attack and environmental cues. Although achievements on understanding the roles of several *KPNβ*s have been obtained from model plant *Arabidopsis thaliana*, comprehensive analysis of potato *KPNβ* gene family is yet to be elucidated. In our genome-wide identifications, a total of 13 *StKPNβ* (*Solanum tuberosum* KPNβ) genes were found in the genome of the doubled monoploid *S. tuberosum* Group Phureja DM1-3. Sequence alignment and conserved domain analysis suggested the presence of importin-β N-terminal domain (IBN_N, PF08310) or Exporin1-like domain (XpoI, PF08389) at N-terminus and HEAT motif at the C-terminal portion in most StKPNβs. Phylogenetic analysis indicated that members of StKPNβ could be classified into 16 subgroups in accordance with their homology to human KPNβs, which was also supported by exon-intron structure, consensus motifs, and domain compositions. RNA-Seq analysis and quantitative real-time PCR experiments revealed that, except *StKPNβ3d* and *StKPNβ4*, almost all *StKPNβ*s were ubiquitously expressed in all tissues analyzed, whereas transcriptional levels of several *StKPNβ*s were increased upon biotic/abiotic stress or phytohormone treatments, reflecting their potential roles in plant growth, development or stress responses. Furthermore, we demonstrated that silencing of *StKPNβ3a*, a SA- and H_2_O_2_-inducible *KPNβ* genes led to increased susceptibility to environmental challenges, implying its crucial roles in plant adaption to abiotic stresses. Overall, our results provide molecular insights into *StKPNβ* gene family, which will serve as a strong foundation for further functional characterization and will facilitate potato breeding programs.

## 1. Introduction

Unlike the prokaryotic ancestors, the nucleus of eukaryotic cells is surrounded by double layers of lipid membranes, called the nuclear envelope (NE), which provides a controlled barrier between nucleoplasm and cytoplasm [1,2,3]. The selective transportation of macromolecules across NE provides the eukaryotic cell with essential and additional benefits in regulating exchange of genetic information in response to the changing environments [4,5,6]. The nucleocytoplasmic transport machinery is composed of a variety of nuclear transport factors: (1) Karyopherin/Importin α (KPN α), which recognize cargo protein with nuclear localization signal (NLS) or nuclear export signal (NES); (2) Karyopherin/Importin β (KPNβ), which binds to KNPα and mediates cargo import into or export out of the nucleus; (3) A small GTPase Ran, which binds to KPNβ and drive directional nucleocytoplasmic transport of cargo-α/β/Ran complex by the RanGTP-RanGDP gradient across the NE [6,7,8,9,10,11]. In addition to collaborate with KPNα in nucleocytoplasmic transport, the KPNβ family of nuclear transport factors can mediate, by directly recognizing NLS/NES with cargos, most macromolecular transport across NE. Therefore, KPNβs are thought to be critical regulators of a set of cellular processes such as signal transduction, gene expression, immune response, etc. [12,13].

KPNβ is typically characterized with an importin-β N-terminal domain (IBN_N, PF03810) or Exporin1-like domain (XpoI, PF08389) at the N-terminus, and a series of tandemly repeated HEAT (Huntingtin, elongation factor 3, protein phosphatase 2A and yeast PI3-kinase TOR1) motifs at the C-terminal portion [13,14]. Based on the evolutionary and transcriptional analysis, KPNβ family is divided into 15 subfamilies which are named according to human nomenclatures [15,16]. Previous experiments have demonstrated that at least 11 human KPNβs and 10 yeast KPNβs can regulate nucleocytoplasmic transport [13].

Members of KPNβ gene family were identified in many eukaryotic organisms from yeast, plant, to mammal. It has been reported that there are 14 members in yeast and over 20 in human genomes, and *Arabidopsis* genome encodes 18 KPNβ proteins, suggesting individual members of KPNβ gene family might have their unique features [12,17,18]. Current knowledge on plant KPNβ genes were mostly obtained from functional analysis of *Arabidopsis* importin mutants [15,19]. For example, AtKPNB1, member of KPNβ1 subfamily, modulates abscisic acid (ABA) signaling and its loss-of-function mutant exhibits enhanced tolerance to dehydration stress due to the increase sensitivity of stomatal closure in response to ABA [20]. PAUSED, an ortholog of human LOS1/XPOT in *Arabidopsis*, is capable of rescuing the tRNA export defect of *los1* in *Saccharomyces cerevisiae* Meyen ex E.C. Hansen, indicating that their functions are highly evolutionarily conserved [21,22]. However, their genomic distribution and biological functions in plant species other than *Arabidopsis thaliana*, to our knowledge, has been largely uninvestigated yet.

Potato (*Solanum tuberosum*), grown on all continents except Antarctica, is the world’s third most important staple crop after rice and wheat in terms of food consumption [23,24,25]. Although most cultivated potatoes are heterozygous autotetraploid and possess the huge genome, wild diploid potatoes with relatively smaller genome become the ideal targets of potato genome sequencing, which could adequately simplify the genome complexity [26]. Furthermore, wild diploid potatoes are widely used as sources of resistance by potato breeders because they are important reserves of genetic and phenotypic variation to biotic and abiotic stresses [27]. The diploid *S. tuberosum* Group phureja DM, cultivated in South America, was chosen to produce a homozygous double-monoploid clone (*S. tuberosum* group Phureja DM1-3 516 R44) using classical tissue culture techniques [25]. The annotated genome of *S. tuberosum* Group phureja DM1-3, was released in 2011 [26], and afterwards draft genome sequence of *Solanum commersonii*, a tuber-bearing wild potato, was also available in 2015 [28,29,30]. The genomic information released facilitates the researches on potato functional genomics, and provides an opportunity to conduct genome-wide analysis of nucleocytoplasmic transporters in potato. Here, we performed a genome-wide, comprehensive analysis of *KPNβ* genes. In total, 13 *KPNβ* genes were identified, and further confirmed by sequencing. The physical and chemical characteristics, genomic structures, chromosomal locations, evolutionary relationship, expression profiles of potato *KPNβ* gene family were analyzed in detail. Finally, VIGS (Virus-Induced Gene Silencing) approach was employed to investigate the role of potato *KPNβ3a*, demonstrating that *KPNβ3a* was associated with plant adaption to salt and oxidative stresses. This study provides the molecular information with respect to the *StKPNβ* gene family, paving the way to the further functional characterization of potato *KPNβ* genes.

## 2. Results 

### 2.1. Genome-Wide Identification of KPNβ Genes from S. tuberosum

To identify KPNβ genes in potato, protein sequences of functionally validated KPNβs from *S. cerevisiae*, *Homo sapiens Linnaeus* and *A. thaliana* were used as the queries to perform BLASTP searches against the potato genome database in Phytozome as well as Potato Genomics Resource. After removing the non-representative splicing forms of same gene locus, 14 KPNβ-like genes were obtained from the genome sequences of *S. tuberosum* phureja DM1-3. Further, the presence of the IBN_N (or XpoI) and Heat repeats domains in these KPNβ-like proteins was scanned using the Conserved Domain Search (CD-search) with e-value <10^-10^. One possible pseudogene (PGSC0003DMG400029568) was removed from our analysis because its expression could not be detected in all samples and conditions examined in subsequent expression analysis, although its protein sequence is identical to *KPNβ3d*. Eventually, only 13 genes were identified as *StKPNβ* genes (Table 1). According to the homologies against *Arabidopsis* and human *KPNβ*s, the nomenclature of these *StKPNβ* genes was listed in Table 1. The predicted proteins encoded by *StKPNβ* varied from 239 amino acids (StKPNβ3c) to 1111 amino acids (StKPNβ3a), with corresponding molecular weights from 27.2 kDa to 123.1 kDa. Of these putative StKPNβ proteins, the theoretical isoelectric points ranged from 4.22 (StPLANTKAP) to 6.10 (StKPNβ3d), indicating that, as weakly acidic proteins, they could participate biochemical processes under disparate in vivo environments. 

### 2.2. Chromosomal Distribution and Duplication Events among StKPNβ Genes

**Figure 1:**
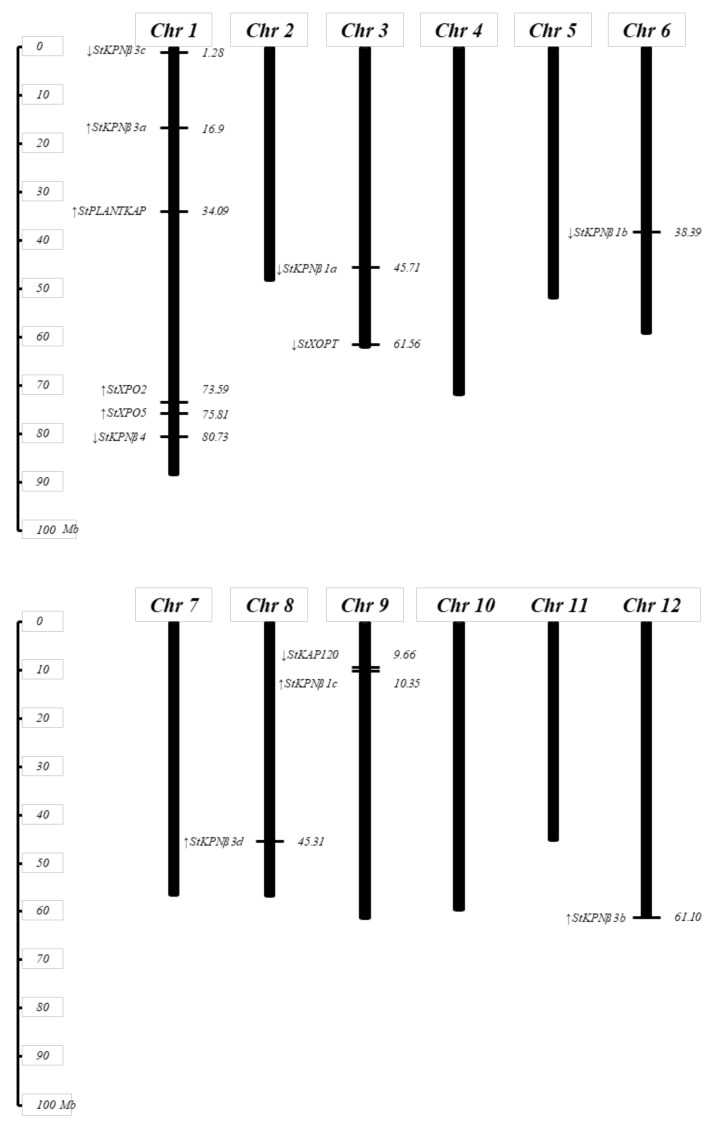
Genomic distribution of *StKPNβ* genes on *S. tuberosum* group phureja DM1-3 chromosomes. The chromosome numbers and size are indicated at the top and bottom of each bar, respectively. The arrows next to gene names show the transcription directions. The number on the right side of the bars designated the approximate physical position of the first exon of corresponding *StKPNβ* genes on potato chromosomes.

The physical map position of *StKPNβ* genes on 12 potato chromosomes was established. The number of *StKPNβ*s are unevenly distributed on the potato chromosomes (Figure 1). Chromosome 1 contains the largest number of *StKPNβ* genes comprising six members, chromosome 3 and 9 each contain two members, whereas chromosome 6, 8 and 12 each contain a single *StKPNβ*. 

The number of *StKPNβ* genes in potato genome is similar to its counterparts in yeast, human and *Arabidopsis*. Pairwise sequence comparison of StKPNβ proteins suggests that the homology broadly ranged from 4.58% (StXPO5 and StXPO2) to 91.71% (StKPNβ1b and StKPNβ1a). Strikingly, through the sequence similarity between StKPNβs, members in two subclades comprising StKPNβ1a/1b/1c share high sequence identity (64.4–91.7%), suggesting that these StKPNβs in KPNβ1 subfamily are likely to be originated from gene duplications while they are positioned to different chromosomes. A similar event was also found in *KPNβ3* subclade, which includes StKPNβ3a/3b/3c/3d with identity from 34.3% to 90.5%. 

### 2.3. Gene Structure of StKPNβs

**Figure 2:**
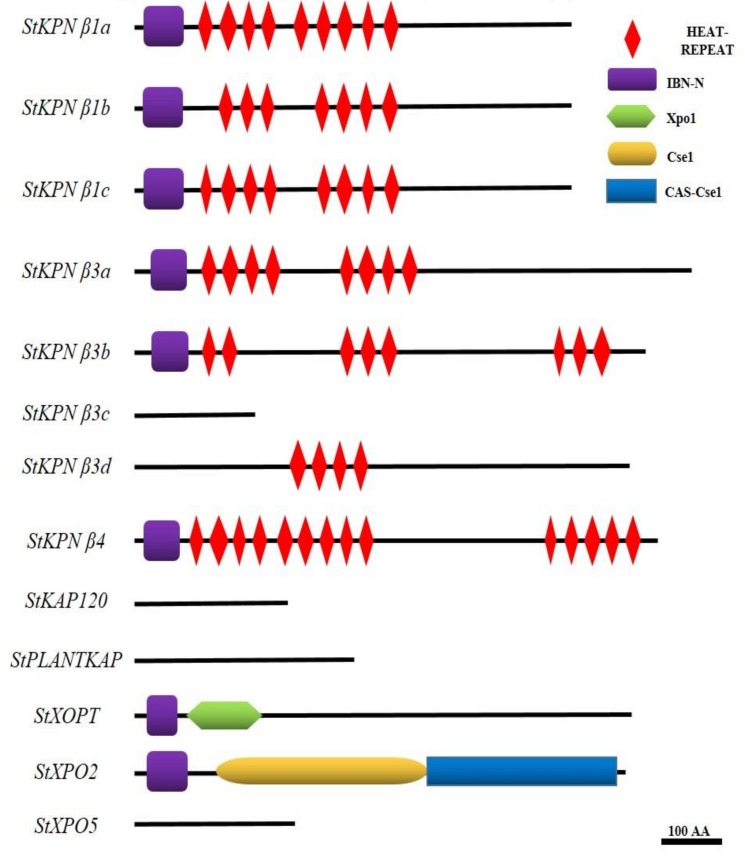
Analysis of conserved domains in StKPNβ proteins. Schematic organization of conserved domains in StKPNβ proteins. The IBN_N domain, HEAT repeats domain, XpoI amd CseI/CAS-CseI domain are shown in purple, red, green and yellow/blue, respectively.

To better understand the gene structure of *StKPNβ*s, the exon-intron features among members of StKPNβ family were aligned via phylogenetic analysis. The phylogenetic analysis revealed three clusters in accordance with the group data presented in Figure 2. Gene structure analysis of all *StKPNβ* genes suggested that the number of exons ranged from 2 to 20, except that *StXPO2* is intronless gene. It is noteworthy that *StKPNβ* members in KPNβ1 subfamily shares identical intron-exon structure. Three members of *StKPNβ3* subclade were also exhibits similar gene structure while *StKPNβ3c* is a truncated gene. Although the exon-intron structure of *StKPNβ*s varies between subclades, it is similar within subclades, which was supported by the phylogenetic analysis of StKPNβ proteins.

### 2.4. Conserved Domains and Motif Analysis of StKPNβs

**Figure 3:**
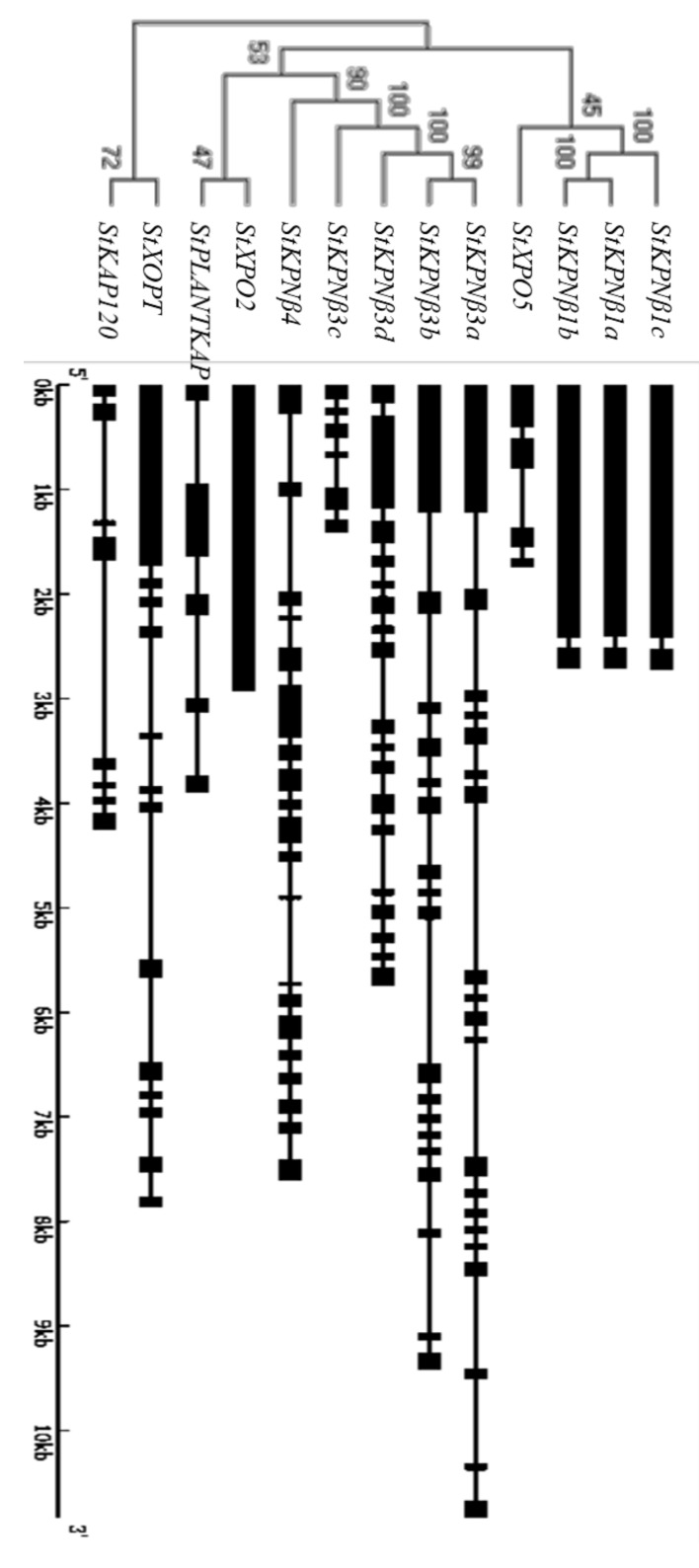
Classification of StKPNβ proteins. Neighbor-joining tree were generated using MEGA X to determine the phylogenetic relationship between StKPNβs (left). The intron-exon organization of *StKPNβ* genes was plotted using Gene Structure Display Server (Version 2.0). Black boxes represent exons and black lines represent introns (right).

It is well-known that members of KPNβ proteins have common features—the IBN_N or XPO1 domains, and HEAT repeats. For members involved export of macromolecules, the conserved region was also called XPO1/CSE1 domains which contain HEAT repeats and a C-terminal domain. To better understand the structural similarity of potato Impβs, we analyzed the amino acid sequences of *StKPNβ* genes using CD-search available at NCBI with default configurations, and re-annotated the domains mentioned above. As shown on Figure 3, eight members of StKPNβs contain both Heat repeat motif and IBN_N motif, and two members possess IBN_N and XPO1/CSE1 domains. There were three potato KPNBs with high sequence similarity to functionally characterized *Arabidopsis* karyopherins, in which no conserved domains were identified by CD-search. StKPNβ3c, homologous to other StKPNβ3 genes, is truncated gene, which resulted in the loss of conserved domains aforementioned. 

**Figure 4:**
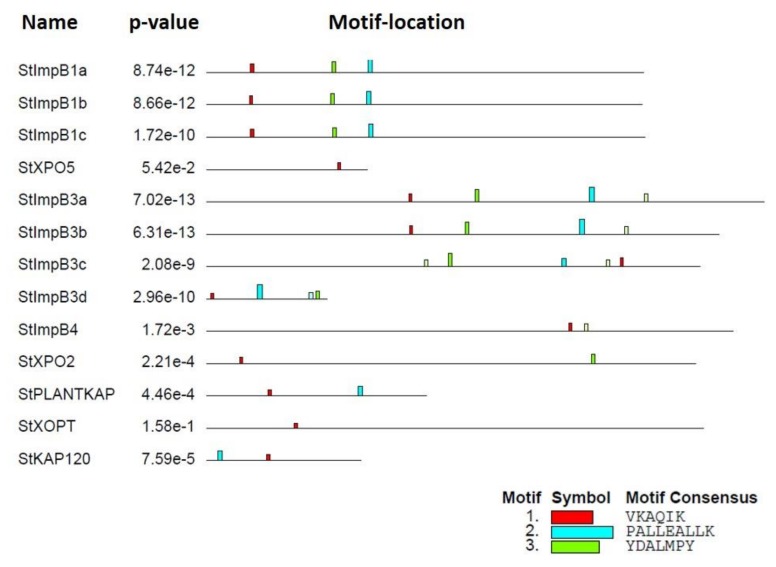
Conserved motifs embedded in the StKPNβ proteins. Conserved motif in StKPNβs was evaluated using the MEME, and the location of novel motifs identified were designated in different colors.

In addition to the HEAT and IBN motifs, we searched the compositions of StKPNβs, which was evaluated using the MEME suite (http://meme-suite.org/tools/meme), an online motif discovery tool. In our analysis, four novel conserved motifs were identified, and among the four motifs, motif I was present in all StKPNβ proteins; Motif II was identified in eight StKPNβ members; and motif III was found in 10 StKPNβs (Figure 4), suggesting that these conserved regions might be essential to execute its biological functions. Furthermore, StKPNβs in the same subfamily shared similar patterns of motif composition, indicating that their functional similarities. Thus, distribution of the motifs also reveals that StKPNβs were likely conserved during the evolution. 

### 2.5. Phylogenetic Analysis of StKPNβs

**Figure 5:**
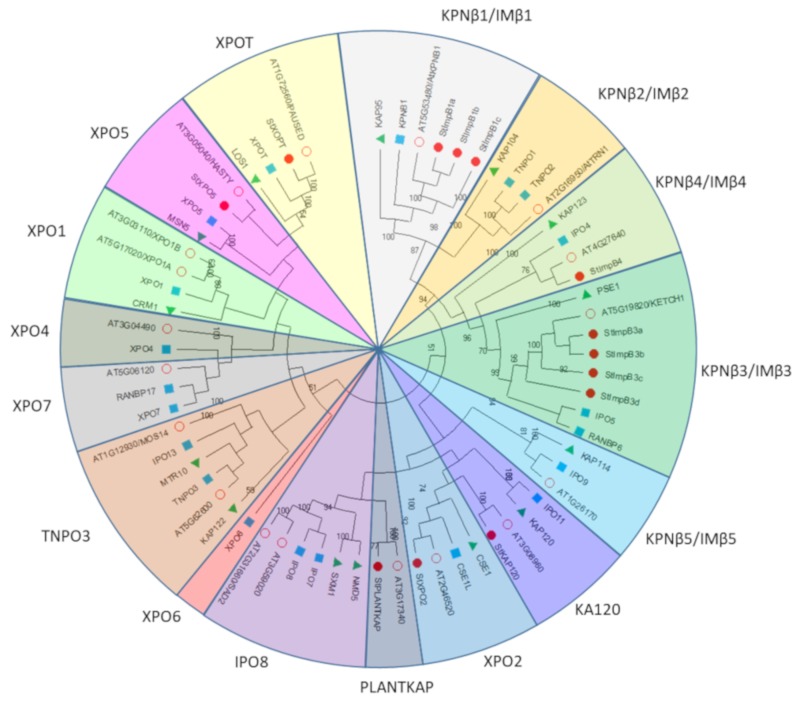
Phylogenetic analysis of StKPNβ proteins in *A. thaliana*, *S. tuberosum*, *S. cerevisiae* and *H. sapiens*. Neighbor-joining tree was constructed based on the alignment of KPNβ protein sequences from *S. cerevisiae* (Green triangle), *H. sapiens* (Blue square), *A. thaliana* (Red empty circle) and *S. tuberosum* (Red circle). The percent bootstrap support for 500 replicates is shown on each branch with >50% support.

To investigate the phylogenetic relationship between the members of *StKPNβ* gene family, a neighbor-joining tree was constructed based on the multiple alignment of karyopherin β protein sequences from *A. thaliana*, *S. tuberosum*, *H. sapiens* and *S. cerevisiae*. All these KPNβ proteins, in accordance with the human KPNβs, were allocated to 16 subfamilies with relatively high confidence (Figure 5). Multiple sequence alignment and phylogenetic analysis suggested that members of the KPNβ family were considerably diverged as the statistical support for some branches was relatively poor. Although yeast is a unicellular organism, at least 13 KPNβs were identified previously in *S. cerevisiae*. These yeast karyopherins included in our analysis actually represented 14 subfamilies of KPNβ nucleocytoplasmic transporters, strongly suggesting that the functional diversification of KPNβ had occurred. Moreover, two yeast KPNβs (NMD5-SXM1), in our phylogenetic tree (Figure 5), were clustered into a sister pair, probably implying they were evolved from a common ancestor. Taken together, the results reinforced that the establishment of KPNβ family predated the appearance of radiation of eukaryote organism, which agrees well with conclusion drawn by O’Reilly et al. [16]. 

As shown on Figure 5, StKPNβs were distributed into two sister pairs of paralogous Impβs (StKPNβ1a/1b/1c, StKPNβ3a/3b/3c/3d) with strong bootstrap support, while the other six form sister pairs with their *Arabidopsis* orthologs. Surprisingly, no potato ortholog could be detected in several KPNβ subfamilies including KPNΒ2/IMB2, KPNΒ5/IMB5, IPO8, XPO1, XPO4, XPO7 and TNPO3, whereas XOPT subfamily is the only one that was lost in *Arabidopsis*. The fact that, compared with yeast and *Arabidopsis*, there are fewer members in the potato KPNβ family reinforces that gene loss occurred after the divergence between Brassicaceae and Solanaceae. Notably, a lineage-specific subclade consisted with two KPNβ members from potato and *Arabidopsis* was detected, suggesting that they might represent a group of plant-specific nucleocytoplasmic transporters. The likely interpretation for the absence of XPO4 and XPO7 subclades from yeast genome indicates that in addition to PLANTKAP subclade, they were derived in multicellular organisms. 

### 2.6. Expression Profiles of StKPNβs among Various Tissues and Developmental Stages

**Figure 6:**
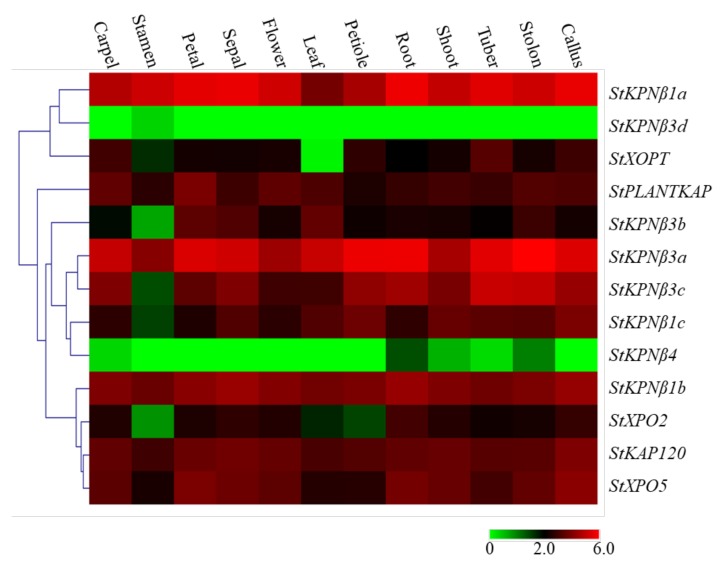
Expression profiles of StKPNβ genes with hierarchical clustering in different tissues. The Illumina RNA-Seq data were obtained from PGSC database, and the FPKM value of representative transcripts of StKPNβs were used to generate heatmap with hierarchical clustering based on the Manhattan correlation with average linkage using MeV software package. Color scale below heatmap shows the expression level; red indicates high transcript abundance while green indicates low abundance.

To gain the insight into the tissue- or organ-specific expression preferences of *StKPNβ* genes, we analyzed the transcriptome data from Illumina RNA-Seq reads generated and stored by PGSC. The transcript abundance of 13 *StKPNβ* genes was determined from the RNA-Seq data as FPKM (Fragments per Kilobase of transcript per Million mapped reads) values. The RNA-seq database was generated from 16 tissues which could be divided into three major groups: floral (carpel, stamen, petal, sepal and mature flower), vegetative (leaf, leaflet, shoot, roots, tuber and stolon) and other tissues (callus) [31]. Digital gene expression analysis revealed that, among these 20 *StKPNβ* genes, *StKPNβ1a/1b/3a*, *StKAP120*, *StXPO5* were ubiquitously and robustly expressed in all tissues, suggesting that these StKPNβs might execute some universal roles and participate nucleocytoplasmic transport in various tissues and organs; conversely, the expression level of *StKPNβ3d* and *StKPNβ4*, compared to other *StKPNβ*s, was relatively lower, suggesting that these KPN-βs might be unnecessary in normal growth conditions (Figure 6). Strikingly, transcripts of *StKPNβ1a/3a/3c* were relatively abundant in tuber or stolon tissues, indicating that their possible association with potato tuber development. These results suggest that, as nucleocytoplasmic regulators, members of StKPNβ family have diverse roles of in potato floral and vegetative tissues.

### 2.7. Expression Profiles of StKPNβs in Response to Biotic and Abiotic Stresses

**Figure 7:**
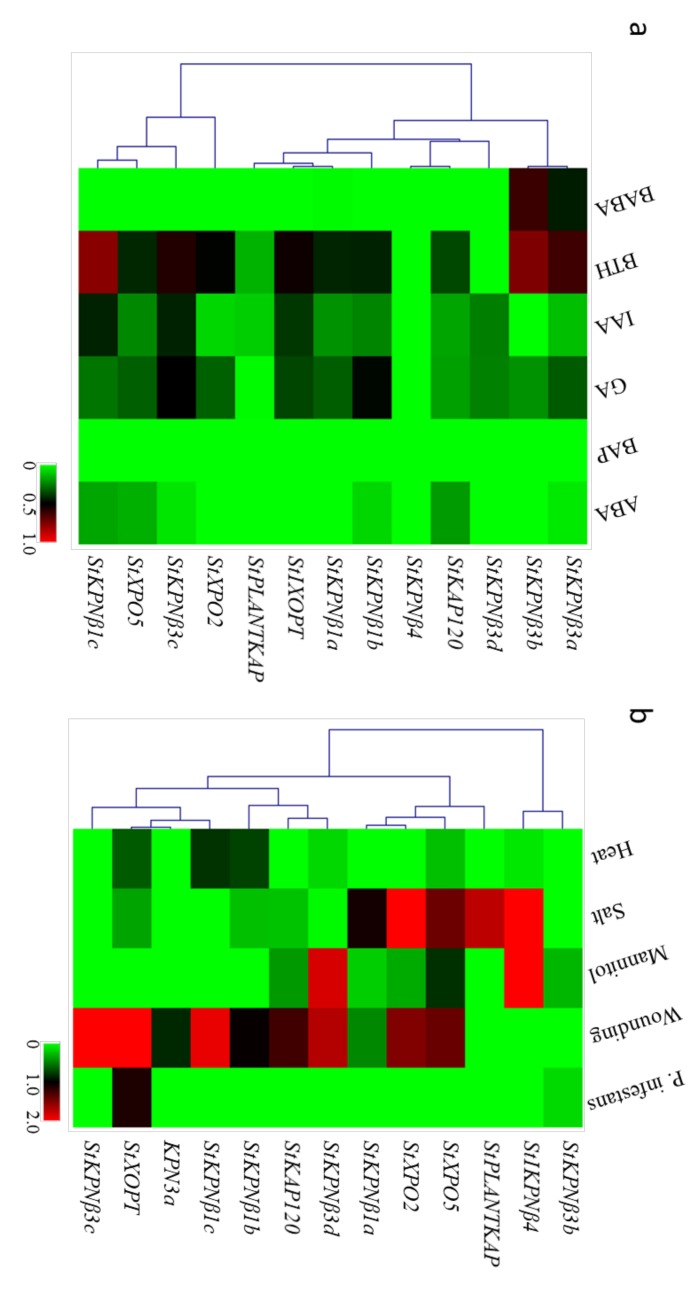
Heatmap representation and hierarchical clustering of *StKPNβ* genes under abiotic and biotic stresses (**a**) and phytohormone treatments (**b**). The Illumina RNA-Seq data were obtained from PGSC database, and the relative expression of *StKPNβ* genes was calculated with respect to control samples using FPKM values of representative transcripts corresponding to *StKPNβ* genes. Fold changes of *StKPNβ* expression were log_2_ transformed, and the normalized expression data was used to generate heatmap with MeV software package using the same parameters in Figure 6. Color scale below heatmap shows the expression level; red indicates high transcript abundance, while green indicates low abundance.

To understand the functions of *StKPNβ* genes under various stresses, the transcript abundance of 13 StKPNβ genes was analyzed the log2 fold change between treatments and controls. RNA-Seq data revealed that most *StKPNβ*s were found to be significantly induced by at least one treatment, while the *StKPNβ3b* transcript was not affected by stress conditions (Figure 7a). Of these 13 *StKPNβ* genes, *StKPNβ4* increased by 2.63-fold under high salinity, and 2.21-fold in response to mannitol stress, while *StKPNβ3d* exhibited a high level of transcription abundance under mannitol and wounding stresses, with 1.90-fold and 1.81-fold increase, respectively. The expression of *StXPO2* and *StXPO5* was increased in response to both salt and wounding treatments. These results suggest that StKPNβs might be serve as core regulators in mediating the signaling transduction of abiotic stresses.

Several *StKPNβ* genes were found to be induced by at least one stress condition (Figure 7a). For example, *StPLANTKAP* were increased by 1.83-fold under salt stress, while in response to wounding treatment, *StKPNβ1c*, *StKPNβ3c* and *StXOPT* were highly increased by 1.94-, 2.15- and 2.30-fold, respectively. The expression specificity of these *StKPNβ*s indicates that they were functionally diverged and actively regulated trafficking of different responsive proteins across the nuclear membrane. It seems that most *StKPNβ*s did not respond to thermal and *Phytophtora infestans* (Mont.) de Bary challenges. The oomycetes *P. infestans* infection resulted in the 1.48-fold expression increase of *StXOPT*, suggesting it might involve the process of plant defense against the pathogen. Therefore, this result suggests that StKPNβs are associated with plant responses to abiotic and biotic stresses.

### 2.8. StKPNβs Response to Various Phytohormones

Similarly, we also examined the expression changes of *StKPNβ*s under different phytohormone or chemical analog treatments by RNA-Seq and quantitative real-time RT-PCR (qRT-PCR) analysis. An interesting observation from RNA-Seq analysis was that expression level of a majority of *StKPNβ* genes were decreased when potato plant being treated with phytohormones or their analogs (Figure 7b). When plants treated with benzothiadiazole S-methyl ester (BTH), a chemical analog of salicylic acid, transcript accumulation of *StKPNβ1c*, *StKPNβ3a* and *StKPNβ3b* genes was observed, suggesting their upregulation possibly contributes to the plant defenses to pathologies. Application of DL-β-amino-n-butyric acid (BABA), known as a disease resistance-priming agent, resulted in the weak induction of *StKPNβ3b.*

Although Illumina RNA-Seq data provides plenty of information on the expression profiles of *StKPNβ* genes, we still lack their expression behavior in response to some important signal molecules such as ethylene (ETH), jasmonic acid (JA), hydrogen peroxide (H_2_O_2_) and salicylic acid (SA). Thus, quantitative real-time RT-PCR (qRT-PCR) analysis was employed to determine the expression patterns of *StKPNβ* genes in these phytohormones or chemicals, and leaf tissues of potato treated with 50 μM SA, 1 mM JA, 1 mM ETH and 50μM H_2_O_2_, respectively, were used in the experiments.

**Figure 8:**
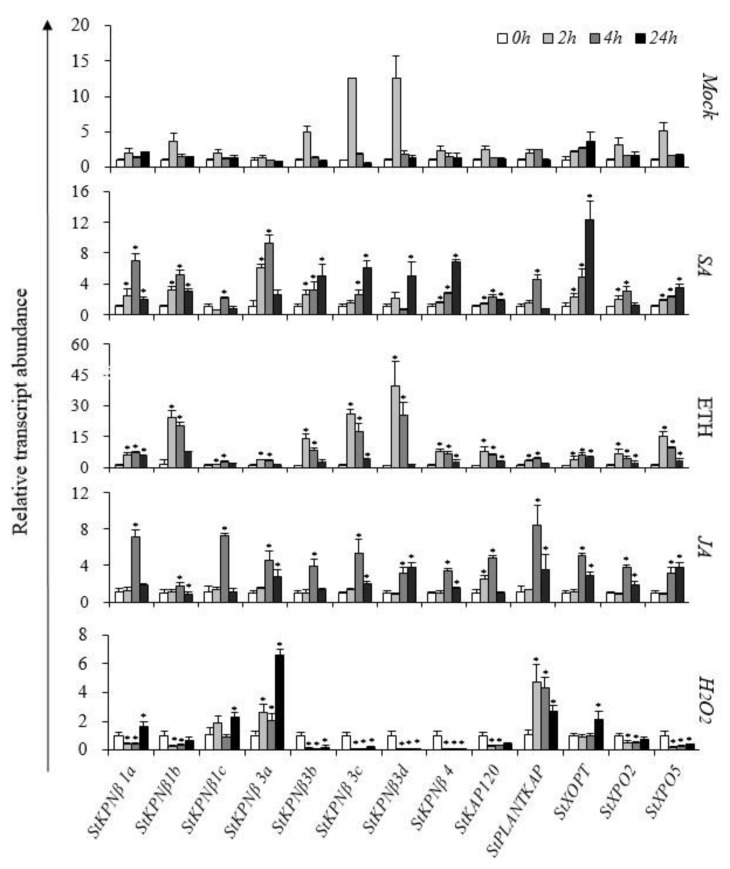
qRT-PCR analysis of *StKPNβ* genes in response to salicylic acid (SA), ethylene (ETH), jasmonic acid (JA) and hydrogen peroxide (H_2_O_2_). *StKPNβ* transcript levels measured by real-time RT-qPCR from the various tissues or under phytohormone treatments at indicated time points. Data are means of three biological replicates (eight pooled plants each), and error bars denote SE. The *StACT* gene was used as an internal control. Stars above the error bars indicate significant differences between treatments and controls (according to student’s t-test). qRT-PCR primers for each *StKPNβ* genes were provided in Table S1.

Most *StKPNβ* genes considered in this study were upregulated upon SA, ETH or JA treatments. Compared to the controls, SA-feeding promoted the expression increase of *StKPNβ1a/3a* and *StXOPT* by at least 6.5-fold under 24 h SA treatment, and similarly *StKPNβ1b/3b/3c/3c/4* and *StPLANTKAP* also exhibited moderately increases, which suggested that they might be involved in the SA-signaling pathway. In JA-feeding experiments, all *StKPNβ* genes displayed an enhanced level of transcript abundance after 4 h treatment, indicating their potential roles in JA-mediated signal transduction. After 4 h ETH treatments, expression of *StKPNβ1b/3c/3d* were strongly activated by ethylene, with 24.0-, 25.8- and 39.9-fold expression increases, respectively; yet, other *StKPNβ*s were slightly induced (Figure 8).

Hydrogen peroxide, predominantly produced during photosynthesis, photorespiration or respiration processes, plays an essential role as signaling molecule in numerous physiological process. The members of *StKPNβ* gene family were simply classified into two groups according to their responsive behavior in response to H_2_O_2_ upregulated and downregulated. The first group represents StKPNβ genes that were induced by H_2_O_2_ and correspond to *StKPNβ1a/1c/3a*, *StPLANTKAP* and *StXOPT*, while the second group includes the remaining *StKPNβ*s, of which the expression negatively responded to H_2_O_2_ (Figure 8). The observations imply that they may be important components of the Reactive oxygen species (ROS) signal cascade in plants. Collectively, these results indicate that StKPNβs were associated with diverse signaling pathways and probably were one of major players in environmental stress and immunity system.

### 2.9. Knockdown of StKPNβ3a Expression Results in Increased Susceptibility to Environmental Stresses

**Figure 9:**
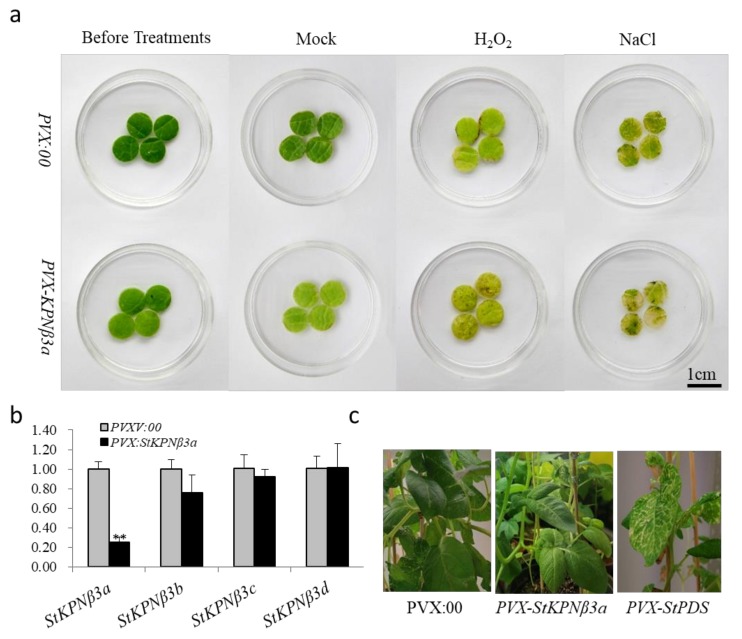
*StKPNβ3a*-silenced potato plants exhibit reduced resistance to salt and H_2_O_2_ treatments. Potato plants were infiltrated with Agrobacterium carrying VIGS-control vector (PVX:00) and PVX-*StKPNβ3a*, and after 2–3 weeks, the *StKPNβ3a-*silencing lines confirmed by qRT-PCR were used for leaf-disk assay. (**a**) Leaf-disk assay for plant tolerance to different abiotic stresses. (**b**) Expression analysis of *StKPNβ3* members in *StKPNβ3a-*silencing and control lines. (**c**) Phenotype of *PVX-StKPNβ3a-*Silencing and control potato plants. The photographs were taken before or after 48-hrs salt (300 mM) or H_2_O_2_ (100μM) treatments, respectively. qRT-PCR analysis of *StKPNβ3a* expression in cotton plants infiltrated with VIGS-control vector (PVX:00) and PVX-*StKPNβ3a*. Error bars indicate SD from three technical replicates of three biological experiments, and asterisks indicate statistically significant differences, as determined by the Student’s t test (**, *p* < 0.01). The experiments were repeated three times with similar results.

Considering that expression of some *StKPNβ* was activated by various stress or hormone treatments, it is plausible that silencing of positively responsive KPNβs would impair the plant tolerance to environmental stresses. Thus, VIGS approach was employed to investigate the role of potato KPNβs. As *StKPNβ3a* was one of highly expressed, H_2_O_2_- and SA-inducible genes, it was chosen for the insertion into the viral vector pGR107 (PVX), and the resulting plasmid *PVX-StKPNβ3a* was introduced into *Agrobacterium* containing the helper plasmid pJIC SA-Rep. The *Agrobacterium* lines harboring *PVX-StPDS* and empty PVX vector (*PVX00*) were served as controls. Potato plants were transformed by leaf-injection with *Agrobacterium* lines aforementioned, and after one month, all silencing lines were verified by qRT-PCR method. We found that leaves of *PVX-StPDS* lines exhibited photo-bleaching phenotypes, which was agreed with the reduction of *StPDS* genes. Compared to the control plants, transcript accumulation of *StKPNβ3a* was decreased in *StKPNβ3a*-sliciencing lines, whereas expression of *StKPNβ3b*, *StKPNβ3c* and *StKPNβ3d* were not significantly affected (Figure 9b), suggesting that *StKPNβ3a* expression was specifically turn down. Under normal conditions, *StKPNβ3a-*sliciencing lines did not exhibit any morphological changes compared to the control plants (Figure 9c). Subsequently, the leaf discs of *StKPNβ3a-*sciliencing as well as experimental controls were floated on the distilled water supplemented with 300 mM NaCl or 100μM H_2_O_2_. After 48-hr salt or H_2_O_2_ treatments, we observed that, compared to the *PVX00* controls, leaf discs of *PVX-StKPNβ3a* lines suffered severe damages (Figure 9a), while there were no evident morphological changes in leaf discs of non-silenced controls. The results illustrated that repression of *StKPNβ3a* could lead to the increased susceptibility to abiotic stresses. 

## 3. Discussion

Karyopherin/Importin β, as an essential nucleocytoplasmic transport receptor, is considered to be a global regulator of diverse cellular functions, ultimately affecting the growth, development and stress adaptions of the eukaryotes [32]. However, current knowledge on its characteristics of was largely obtained from functional characterization of animal and yeast *KPNβ* genes. In the past two decades, achievements have been made in understanding the role of KPNβ in model plant *A. thaliana*, and several *KPNβ* genes, including *Hasty*, *SAD2/EMA1*, *AtKPNB1*, *MOS14* and *KETCH1*, were investigated in detail, demonstrating their vital roles involved in the *Arabidopsis* development, biotic and abiotic stresses [21,22,33,34,35,36]. However, the identification and functional analysis of KPNβ homologs still limited in plants other than *Arabidopsis*. Hence, analyses of *KPNβ* gene family in *S. tuberosum* become indispensable in understanding of its gene structure, protein function and evolution. 

The number of *KPNβ* genes varies among organisms. In the study, 13 *KPNβ* genes were identified from potato genome, whereas previous search identified 18 *KPNβ*s in *Arabidopsis* [20,31]. Considering that potato has undergone two rounds of whole-genome duplication (WGD) events so that the genome size of DM1-3 potato was nearly five times larger than *Arabidopsis* [26], the observations on *StKPNβ* gene family contradicted with genome complexity between potato and *Arabidopsis*. Therefore, it is interesting that the number of *StKPNβ* genes was much less than that of *Arabidopsis*. Our phylogenetic analysis revealed that eight KPNβ subfamilies, namely KPNβ1/IMβ1, KPNβ3/Impβ3, KPNβ4/Impβ4, KA120, PLANTKAP, XPO2, XPO5 and XOPT, were represented by at least one KPNβ ortholog in potato genome, and duplication events occurred only in KPNβ1 and KPNβ3 subfamilies, perhaps due to the independent, small-scale, segmental duplication events and chromosome rearrangements in the two loci. Nevertheless, in comparison to yeast and *Arabidopsis*, it seems that homologs to other seven KPNβ subfamilies (KPNβ2/Impβ2, KPNβ5/Impβ5, IPO8, XPO1, XPO6, XPO4, XPO7 and TNPO3) were lost completely during the evolution in potato genome, consequently resulting in the fewer members of KPNβ in potato genome.

Functional redundancy and diversification were observed in potato KPNβ1/Impβ1 and KPNβ3/Impβ3 gene subfamilies. With respect to KPNβ1 subfamily, phylogenetic analysis and sequence alignment revealed the existence of three genes, namely *StKPNβ1a*, *StKPNβ1b* and *StKPNβ1c*, homologous to *AtKPNB1*, which raises the possibility that these *StKPNβ1*s might execute similar functions. Consistent with the assumptions, we found that expression patterns under stress or phytohormone treatments, to a large extent, resembled among members of *StKPNβ1*, implying that members of *StKPNβ1s* might share some conserved and overlapping functions. Nevertheless, it was noteworthy that some expression discrepancies between *StKPNβ1* genes, because expression analysis demonstrated that only *StKPNβ*1a could not respond to wounding stress, while expression of *StKPNβ*1c, instead of *StKPNβ*1a and *StKPNβ*1b, was able to be strongly activated by wounding treatment, which reflects that members of *StKPNβ*1 subfamily might have acquired its unique roles through functional diversifications. 

Recent investigations have reported that a few *Arabidopsis* KPNβ/Impβs, as nucleo-cytoplasmic transport receptors, are involved in stress adaption under abiotic and biotic stresses, while they are not stress-inducible genes [20,37]. *AtKPNB1* encodes an ortholog of human KPNB1 in *Arabidopsis*, and *kpnb1* loss-of-function mutant exhibits increased sensitivity to ABA [20]. It was proven that AtKPNB1, functioning as negative regulator, could regulate the ABA responses and drought tolerance via ABI1- and ABI5-independent pathways, though ABA treatment only slightly boosted the transcript accumulation of *AtKPNB1* [24]. In addition, absence of SAD2 (Super sensitive to ABA and Drought 2), member of IPO8 subfamily, led to the enhanced sensitivity to ABA, H_2_O_2_ or drought in *Arabidopsis*, whereas its expression was independent from phytohormone or stress treatments [37,38]. In agreement with previous findings, our RNA-Seq analysis also suggested that many members of *StKPNβ* gene family did not show any transcriptional responses to hormones or stresses examined, and only several *StKPNβs*, such as *StKPNβ3d, StKPNβ3b, StPLANTKAP, StXOPT* etc., were able to respond to environmental cues or phytohormone inductions. 

It is tempering to analyze the roles of responsive StKPNβs, especially whose expression could be activated by hormones, environmental cues or pathogen infections. Expression of *StKPNβ3a* was strongly induced by SA, JA or H_2_O_2_ treatments, suggesting its involvements in the phytohormone cascades. Thus, using VIGS approach, we demonstrated that silencing of *StKPNβ3a* resulted in the increased susceptibility to salt or oxidative stresses, supporting that its function is indispensable in the stress signaling transductions. However, due to the lack of stable transgenic lines of *StKPNβ3a*-overexpression or -RNAi, the biological functions of *StKPNβ3a* still need to be investigated in detail. Phylogenetic analysis supported that *StKPNβ3a* were orthologous to yeast *PSE1/Kap121*, human IPO5 and RANBP6. Yeast strains with disruption of PSE1 functions exhibit delayed mitosis and enhance sensitivity to temperature stress, while overexpression *PSE1* contributes to the three-fold increase of cellulose production [39,40,41]. The import of histone H2A/H2B and H3/H4 is mainly mediated by PSE1 in *S. cerevisiae*, suggesting its essential roles in intranuclear transport [42,43]. It has been demonstrated that human IPO5 also functions in the nuclear import of essential histones as well as some ribosomal proteins [44]. Given that members of *Impβ3* subclades play key roles in nucleocytoplasmic trafficking, it is reasonable that *StKPNβ3a* might execute the similar roles by regulating the import of positive regulatory protein(s) under abiotic stresses. Further investigations will be still required to identify its cargo(s) and to articulate the molecular mechanism of Impβ-mediated signaling pathway in plants.

## 4. Materials and Methods

### 4.1. Plant Material and Treatments

*S. tuberosum* Phureja DM1-3 or cultivar “Shpedy” plants were in vitro micropropagated on Murashige and Skoog (MS) medium plus 30 gL^−1^ sucrose and 0.8% agar (Sigma-Aldrich, USA), with pH adjusted to 5.8. Potato seedlings were routinely subcultured as two-node segments every 3–4 weeks and incubated at 23 °C with 16 h photoperiod under cool with fluorescent lamps (~70 μmol m^−2^ s^−1^ photon flux idensity). 3-week old potato plants were subjected to IAA (50 μM), SA (1 mM), ethylene (1mM) or H_2_O_2_ (1mM) treatments. The plant tissues were collected at designated points and immediately frozen in liquid nitrogen. Sample collections were performed on separate days for the replicates.

### 4.2. Identification of KPNβ Genes in S. tuberosum Group Phureja

To investigate the KPNβ gene family in in *S. tuberosum* Group phureja DM1-3, all members of KPNβ/Impβ sequences from Human (*H. sapiens*), yeast (*S. cerevisia*e) and *Arabidopsis* were used as queries for BLAST search against Phytozome (https://phytozome.jgi.doe.gov/), NCBI (http://blast.ncbi.nlm.nih.gov/), Potato Genomics Resource (http://solanaceae.plantbiology.msu.edu/) and other online resources with default parameters. The StKPNβ candidates were confirmed the presences of IBN_N (PF08310) or XpoI (PF08389) domain, and HEAT repeats using SMART (http://smart.embl-heidelberg.de/smart/batch.pl) and CDD-search. In order to obtain non-redundancy KPNβ sequences, potato KPNβ sequences were used as queries to blast against Phytozome database, and any redundancy was manually removed. The representing gene model per *StKPNβ* locus were identified and their corresponding information on chromosomal location, locus ID, transcript ID were obtained simultaneously.

### 4.3. Analysis of Gene Structure and Conserved Domains

Based on the genome annotation of DM assembly available in Phytozome, the intron-exon structure of individual *StKPNβ* genes was predicated, and its genomic organization was visualized using Gene Structure Display Server 2.0 (GSDS, http://gsds.cbi.pku.edu.cn/) [45]. Conserved domains in protein sequences were verified using ScanProsite (http://pro-site.expasy.org/scanprosite/), which provides information about positions of different domains in the protein sequence. This information was used to draw visual representation of distribution of domains in the deduced amino acid sequences of proteins using Microsoft Office PowerPoint 2016.

### 4.4. Sequence Alignment and Phylogenetic Construction

Multiple alignment of KPNβ protein sequences from *A. thaliana*, *S. tuberosum*, *H. sapiens* and *S. cerevisiae* was conducted using ClustalW [46]. Neighbor-joining method was used to conduct a phylogenetic tree analysis in MEGA X, with 500 bootstrap replicates and randomized sequence input order.

### 4.5. Expression Profiling of StKPNβ Genes in Different Tissues or Under Various Stresses

The RNA-Seq data corresponding to *StKPNβ* genes was downloaded from the Potato Genomics Resource [31], and the corresponding FPKM (fragments per kilobase per million reads) values for *StKPNβ* genes were obtained for 12 tissues representing major organs and developmental stages, including floral (carpel, petals, sepals, stamens and mature flower), leaf (whole leaf, leaflet and petiole), tuber (tuber and stolon), and other organs (shoot, root and callus). As described, biotic and abiotic treated tissues included potato plants exposed to heat (35 °C), NaCl (150 mM) or Mannitol (260 mM), and leaves challenged by *P. infestans*, BABA (DL-β-amino-n-butyric acid), BTH (6-benzylaminopurine) or hormones [31]. Similarly, FPKM values for abiotic or biotic stress-treated potato plants were analyzed by calculating the fold change of expression levels between treatments and the corresponding controls. The normalized expression data was used to generate heatmap by using the MeV software package (http://mev.tm4.org) available at the Institute for Genomic Research, and hierarchical clustering analysis (HCA) was built on the basis of the Manhattan correlation with average linkage method. 

### 4.6. RNA Extraction and Quantitative Real-Time RT-PCR

Total RNA was extracted with Trizol (Invitrogen Inc., Madison, WI, USA) as described previously [47,48]. RNA quantity and quality were assessed using a NanoDrop8000 (Thermo Scientific™, Wilmington, DE, USA). Total RNA isolation and reverse transcription with oligo (dT)_18_ (18418-012; Invitrogen, Madison, WI, USA) were performed as described previously. The amounts of individual genes were measured with gene-specific primers by real-time PCR analysis with a cycler IQ real-time PCR instrument CFX96 and SYBR Green mixture (Bio-Rad, Foster City, CA, USA). The relative expression of specific genes was quantitated with the 2^−∆∆*C*t^ calculation method [49], where ΔΔCt is the difference in the threshold cycles and the reference housekeeping gene, which was potato *StACT* (PGSC0003DMG400027746) for expression analyses. The sequences of specific primers are shown in Table S1.

### 4.7. Virus-Induced Gene Silencing (VIGS) of Potato

The potato virus X (PVX)-induced gene silencing is conducted as described previously [50,51]. Briefly, *PVX-StKPNβ3a* were generated by cloning a PCR fragment amplified by *S. tuberosum* phureja DM1-3 potato leaf cDNA template using specific oligonucleotide primers incorporating *SalI* and *ClaI* restriction sites, respectively, at the 5′- and 3′-ends for cloning into virus vector pGR107. The *Agrobacterium tumefaciens* (Smith & Townsend, 1907) strain GV3101 harboring the recombinant plasmids *PVX*-*StKPNβ3a* and help plasmid pJIC SA_Rep were used for in vitro agroinoculation by leaf-injecting of 4-week-old potato plants. The *Agrobacterium* lines carrying with *PVX-StPDS* and the PVX vectors were used as positive and negative controls, respectively. Primers used for RT-PCR amplifications are listed in Table S1.

## 5. Conclusions

In this study, the systematic characterization of *KPNβ/Impβ* gene family was performed in the *S. tuberosum*. A total of 13 *StKPNβ* genes were identified through searching potato genome, and their chromosomal distribution, conserved domain, motif composition and intron-exon structure were studied in detail. Expression analysis based on the RNA-Seq and qRT-PCR analysis suggested that several *StKPNβs* was responsive to biotic and/or abiotic stresses. Furthermore, the function of *StKPNβ3a* was characterized through VIGS approach, illustrating that it might be a promising candidate gene for molecular breeding. In summary, our results provide valuable insights of *StKPNβs* gene family, which will facilitate further functional analysis of *StKPNβs* and will also benefit genetic engineering of potato. 

## Figures and Tables

**Table 1:** List of putative *StImpβ* gene family members of *S. tuberosum* Group phureja.

Gene Name ^a^	Locus ID ^b^	Predicted Proteins	Chromosomal Location ^c^			Gene Models ^d^	Putative Proteins ^e^		
			Chr	Chr_start	Chr_end		Length (aa)	pI	MW (kDa)
*StKPNβ 1a*	PGSC0003DMG400018525	PGSC0003DMP400032281	3	45710853	45713572	1	871	4.61	96.40
*StKPNβ 1b*	PGSC0003DMG400019597	PGSC0003DMP400034029	6	38386746	38389457	1	868	4.59	96.00
*StKPNβ 1c*	PGSC0003DMG400026641	PGSC0003DMP400046282	9	10350557	10353269	1	873	4.62	96.22
*StKPNβ 3a*	PGSC0003DMG400015862	PGSC0003DMP400027802	1	16901021	16911855	1	1111	4.74	123.08
*StKPNβ 3b*	PGSC0003DMG401004281	PGSC0003DMP400007618	12	61091176	61100591	1	1021	4.77	113.87
*StKPNβ 3c*	PGSC0003DMG400023766	PGSC0003DMP400055376	1	1281756	1283167	1	239	4.73	27.15
*StKPNβ 3d*	PGSC0003DMG400013325	PGSC0003DMP400051493	8	45306052	45311794	1	983	6.10	109.56
*StKPNβ 4*	PGSC0003DMG400032173	PGSC0003DMP400041127	1	80738183	80745787	5	1049	4.87	115.27
*StKAP120*	PGSC0003DMG401000117	PGSC0003DMP400011095	9	9658233	9662484	1	307	5.28	33.77
*StPLANTKAP*	PGSC0003DMG400006259	PGSC0003DMP400021286	1	34095216	34099108	1	438	4.22	49.23
*StXOPT*	PGSC0003DMG400012034	PGSC0003DMP400039669	3	61569820	61577676	4	990	5.44	111.51
*StXPO2*	PGSC0003DMG400022883	PGSC0003DMP400000259	1	73593165	73596092	1	975	5.52	109.63
*StXPO5*	PGSC0003DMG400022491	PGSC0003DMP400038992	1	75806958	75808695	1	320	6.00	35.01

^a^ Name referred to systematic designation to members of *KPNβ* family in *S. tuberosum* according to the homology against *Homo sapiens*. ^b^ Gene accession number in PGSC database. ^c^ Chromosomal location of the St*KPNβ* genes in the DM1-3 potato genome (V4.3). ^d^ isomer numbers. ^e^ Length (number of amino acids), molecular weight(kilodaltons), and isoelectric point (pI) of the deduced polypeptides were calculated using Lasergene Molecular Biology Suite (Version 7.0).

## Supplementary Materials

Supplementary materials can be found at https://www.mdpi.com/1422-0067/21/3/931/s1.
