# Supplementary material for: Genome-Wide Identification, Expression Profile and Evolution Analysis of Karyopherin β Gene Family in Solanum tuberosum Group Phureja DM1-3 Reveals Its Roles in Abiotic Stresses
Source: Int J Mol Sci. 2020 Jan 31;21(3):931. doi: 10.3390/ijms21030931 (PMC7037939; doi:10.3390/ijms21030931)
Supplement: Supplementary file 1 [file ijms-21-00931-s001.pdf]

International Journal of *Molecular Sciences*

MDPI

Table S1. Primers used in qRT-PCR and VIGS experiments.

No.	Primer name	sequences(5' to 3')
1	StKNPβ 1a-qRT-F	ATGACAGAGCTGCACCAGAC
	StKNPβ 1a-qRT-R	GGCTCGGAAGCCCCTAATTT
2	StKNPβ 1b-qRT-F	AAGATGCTGGTGAGTCACGG
	StKNPβ 1b-qRT-R	CATCGGTCGAACACCTCACA
3	StKNPβ 1c-qRT-F	GTTGCCAAAGACAGCCCAAG
	StKNPβ 1c-qRT-R	TGCACTGGAACCAAGAGCAT
4	StKNPβ 3a-qRT-F	ATTGCTCAGGGCCGAAATGA
	StKNPβ 3a-qRT-R	TCGGACCTGACCTTCATCCA
5	StKNPβ 3b-qRT-F	CAGCTTTGGTGGAAGCTTTG
	StKNPβ 3b-qRT-R	ATTCACCTTCCTCGGCATCA
6	StKNPβ 3c-qRT-F	TGCGTTGCAAAAAGTGAGCA
	StKNPβ 3c-qRT-R	ATGCTCTGCACCTGACCTTC
7	StKNPβ 3d-qRT-F	ATGGCAAGTCCCCAGTTCAA
	StKNPβ 3d-qRT-R	GAGCATCTGGAGAGGACCTT
8	StKNPβ 4-qRT-F	CTCAACACATGGGTGCTCCT
	StKNPβ 4-qRT-R	TTCGGTTTGTAGCATCCGCT
9	StKAP120-qRT-F	GTTTCCCGATGGAAGTGCCT
	StKAP120-qRT-R	ATGATCATCTCCCCCGGTCA
10	StPLANTKAP-qRT-F	ACAGCATGCGTGGCTAAGAT
	StPLANTKAP-qRT-R	ACATGATGAAGGGGGCACTG
11	StXOPT-qRT-F	TGTAACTGCCAGTCGTCCTG
	StXOPT-qRT-R	ATGTCCACCATGCGGTGAAT
12	StXPO2-qRT-F	TCCGCAAATGTTCCAGCTCT
	StXPO2-qRT-R	TGCTGGTTAAGCTCATGGGG
13	StXPO5-qRT-F	TTGGCTGCATTTGCCACAAG
	StXPO5-qRT-R	TGCAAGGCTGGAAGTGCTAT
14	StAct-qRT-F	CGACCACCTTAATCTTCATGC
	StAct-qRT-R	TACTCGTTCACCACCTCAGC
15	StKNPβ 3a(ClaI)-VIGS-qRT-F	ATCTTCTCTCCTCTTCACCTCACCA
	StKNPβ 3a(SalI)-VIGS-qRT-R	AGGACTGATTTGATACCTGACTGAG
16	StPDS-qRT-F	AAAGGTACCTTGAATGAGG
	StPDS-qRT-R	AAAGAGCTCAATGGCCGAC
